# The network structure of ICD-11 adjustment disorder: A comparison of clinical and nonclinical samples

**DOI:** 10.1192/j.eurpsy.2022.2303

**Published:** 2022-07-29

**Authors:** Yafit Levin, Thanos Karatzias, Mark Shevlin, Menachem Ben-Ezra, Andreas Maercker, Rahel Bachem

**Affiliations:** 1 Education Department, University of Ariel, Kiryat HaMada 3, Ariel 40700 Israel; 2 School of Social Work, University of Ariel, Ariel, Israel; 3 Edinburgh Napier University, Sighthill Ct, Edinburgh, Scotland EH11 4BN, United Kingdom; 4 NHS Lothian, Rivers Centre for Traumatic Stress, Edinburgh, Scotland, United Kingdom; 5 School of Psychology, Psychology Research Institute, Derry, Northern Ireland; 6 Institute of Psychology, Psychopathology and Clinical Intervention, University of Zurich, Binzmühlestrasse 14, Zurich 8050, Switzerland

**Keywords:** Adjustment disorder, ADNM-8, ICD-11, symptoms network analysis

## Abstract

**Background:**

International Classification of Diseases, 11th revision (ICD-11) adjustment disorder (AjD) is characterized by two main symptom clusters: preoccupation with the stressor and failure to adapt to the stressor. The network analytic approach provides important information on the structural validity of a disorder and reveals which symptoms are most prominent. To date, no study compared the network structure of AjD symptoms in clinical and nonclinical samples, which could potentially inform our understanding of psychopathological mechanisms that underlie AjD and identify core targets for therapy.

**Methods:**

A network analysis was conducted on AjD symptoms as assessed by the Adjustment Disorder—New Module (ADNM-8) using data from 330 clinical participants from the UK and a nonclinical sample of 699 participants from Switzerland.

**Results:**

Comparisons of network structure invariance revealed differences between the network structure of the clinical and the nonclinical samples. Results highlight that in terms of both edges strength and centrality, failure to adapt symptoms was more prominent in the clinical sample, while the preoccupation symptoms were more prominent in the nonclinical sample. Importantly, global strength was similar across networks.

**Conclusions:**

Results provide evidence of the coherence of AjD in the ICD-11 as assessed by the ADNM questionnaire. They tentatively suggest that subclinical AjD may be characterized by emerging preoccupation symptoms that may result in failure to adapt and functional impairment in clinical manifestation of AjD. However, there is a need for replication and longitudinal research to further validate this hypothesis.

## Introduction

Adjustment disorder (AjD) is one of the most frequently diagnosed mental health conditions in clinical practice [1, 2] and is prevalent in the general population. For example, 15.6% of participants in a nationally representative sample of Ireland screened positive for AjD [3], whereas 16.5% in the general population of Lithuania fulfilled the diagnostic criteria [4]. Even though AjD is defined as a self-resolving condition, it can be protracted if the stressor continues, resulting in a substantial decline in quality of life and an increased risk of suicide [5, 6]. Recently, the International Classification of Diseases, 11th revision (ICD-11) [7] has revised the diagnostic conceptualization of AjD and for the first time represents it by specific symptom groups.

According to ICD-11, AjD is a maladaptive reaction to a stressful life event, ongoing psychosocial adversities, or a combination of stressful life situations that usually emerges within a month of the occurrence of a stressor and tends to resolve within 6 months, unless the stressor persists for a longer duration. In ICD-11, AjD is characterized by two main symptom clusters: “preoccupations with the stressor,” which includes symptoms such as recurrent and distressing thoughts or rumination about the stressor or its implications, and “failure to adapt,” which includes difficulties concentrating, sleep disturbances, and an inability to recover emotionally. For a diagnosis of AjD, the symptoms must be associated with significant impairment in functioning [7].

In parallel to the development of the AjD symptom criteria, a scale to assess AjD has been developed for validation of the newly proposed concept. Maercker et al. [8] introduced and initially validated a 29-item self-report questionnaire, the Adjustment Disorder–New Module (ADNM), which was later condensed to 20 items [9]. The ADNM-20 can be used to assess the two core symptom clusters of AjD in ICD-11 (preoccupation with the stressor and failure to adapt). Several validation studies of both ADNM versions indicated good psychometric properties [10, 11]. More recently and in line with the conceptualization of AjD in the ICD-11, an 8-item brief version, consisting of only the core symptoms [12], was produced and validated.

Factor analytic models assume a predetermined set of factors [13] which means they are less efficient in providing the full complexity of relations among the different symptoms of AjD. The network approach, on the other hand, conceptualizes mental disorders as systems of connected symptoms rather than reflecting an unobservable disorder. A network structure consists of “nodes” that represent the symptoms studied and edges that represent the relationship between nodes. Edges have thicknesses corresponding to the strength of the association between the nodes they connect [14]. The symptoms co-occur because they reciprocally reinforce each other, not because they arise from a common underlying cause [13].

Another advantage of the network approach is the index of central symptoms which are having many strong connections to other symptoms and greater numbers of connections [15]. Identifying central symptoms of a disorder is of crucial importance to clinicians in order to guide intervention efforts. Central symptoms can also guide prognosis of patients and inform the development of care plans accordingly. Preliminary findings suggest that symptom centrality is related to the longitudinal course of a disorder [16]. In the case of AjD, very few disorder-specific interventions have been developed to date [17] and thus, obtaining information on symptom centrality may be particularly relevant for improving future treatment efforts in clinical samples.

To the best of our knowledge, no study has yet examined the network analysis of AjD in a clinical sample. The network of AjD was examined recently for the first time, in the general population of three African countries, and revealed important insights into the complex relations among its symptoms [18]. Results highlighted preoccupation symptoms as the more prominent symptoms in terms of edges strengths and had the highest centrality in all networks. Scrutinizing the nature of both preoccupation and failure to adapt symptoms and their importance in clinical populations as compared to general population samples could help us to understand psychopathological mechanisms that underlie AjD and broader psychopathologies.

Furthermore, while the new conceptualization of AjD in ICD-11 suggests a two-factor structure [18], there is evidence to suggest that AjD could be perceived as a unidimensional construct [19]. It would be of interest to examine the differences in network structure between clinical and nonclinical samples and the pattern of connections across different symptoms. The first network analyses have been performed among nonclinical samples and that therefore, the analysis should be performed on a clinical sample to identify the organization of the symptoms within a clinical sample. The current study thus aimed to compare the network structure of AjD in a clinical sample and a nonclinical sample.

We aimed to explore whether the networks are different in terms of global strength and structure. We aimed specifically to compare networks on (a) conceptual validity by exploring which of the symptoms are strongly associated with one another and are located adjacently; (b) which symptoms are most central and whether they belong to the preoccupations or the failure to adapt clusters.

## Methods

### Participants and procedure

The study sample included 330 participants from Scotland (*n* = 330) and Switzerland (*n* = 699).

#### Clinical: UK sample

Data were collected from a trauma clinic as part of routine initial assessments (*n* = 330 participants). The clinic is receiving referrals from GPs, psychiatrists, and other mental health services of people who have experienced psychological trauma. Individual and group treatments for psychological trauma are being offered by qualified therapists. Participants were a consecutive sample of adults who self-referred to an NHS trauma service in Scotland (*N* = 330). All new patients over the 8-month recruitment period were asked to complete a set of standardized measures as part of their initial assessment with the service. Eligibility criteria for participation were as follows: having self-referred to the service for psychological therapy within the recruitment period, being aged 18 years or over, and possessing adequate competency in written English to allow for the completion of self-report questionnaires. Ethical approval for the collection and use of these data was provided by NHS Lothian Clinical Governance and Edinburgh Napier University Research Ethics Committee. The mean age of the participants was 38.97 years (*SD* = 12.46, range 18–78 years), and 62.1% were female (*n* = 205). Almost the entire sample was of British ethnicity (*n* = 297, 92.5%) while 4.7% (*n* = 15) were from other European nations, and 1.2% (*n* = 4) were Asian. Less than half of the sample was employed at the time of assessment (40.0%, *n* = 132). In addition, 6.7% were students (*n* = 22), 7.8% (*n* = 25) were home keepers, 29.8% (*n* = 95) were unemployed or retired, 9.1% (*n* = 29) were not working due to illness, and 1.4% (*n* = 13) were retired. The majority of the sample were not having an in-patient care history (*n* = 279, 87.2%), while 12.8% (*n* = 41) had an in-patient care history.

The entire sample endorsed the full criteria of AjD according to ICD-11 as tested by the ADMN-8. The most frequently endorsed stressful life events were family conflicts (58.5%), financial problems (50.5%), and too much/too little work (48.0%). See Table 1 for more information.

**Table 1.** Prevalence of stressors in the clinical sample.

Stressful life events	*n* (%)
Family conflicts, yes	176 (58.5)
Financial problems, yes	152 (50.5)
Too much/too little work, yes	144 (48.0)
Termination of an important leisure activity, yes	150 (45.4)
Illness of a loved one, yes	124 (41.2)
Own serious illness, yes	121 (40.2)
Pressure to meet deadlines/time pressure, yes	113 (37.5)
Death of a loved one, yes	109 (36.2)
Unemployment, yes	107 (35.5)
Moving to a new home, yes	105 (34.9)
Divorce/separation, yes	91 (30.2)
Conflicts in work-life relations, yes	88 (29.2)
Assault, yes	76 (25.2)
Conflicts with neighbors, yes	49 (16.3)
Serious accident, yes	32 (10.6)
Adjustment due to retirement, yes	9 (3.0)

#### Nonclinical: Switzerland sample

Participants (*N* = 699) consented to participate in a study aiming to uncover psychosocial coping with challenges regarding COVID-19. We considered the pandemic as a global stressor which satisfies the criteria for exposure to a stressful life event that could potentially trigger AjD, as has been suggested in previous research [20, 21]. The study was approved by the Institutional Review Board of the University of Zurich. Data collection took place from April 24 to May 23 while Switzerland was in a partial lockdown. Inclusion criteria were being above the age of 18 and being fluent in German. Participants were recruited via social media (e.g., Facebook), using a snowball technique. The study was advertised through personal and professional networks and included a Facebook advertisement targeting ages 30 and above. Questionnaires were distributed electronically in German using Unipark Software. The participants provided their written informed consent to participate in this study. The mean age of the participants was 43.45 years (*SD* = 15.09, range 18–87 years), and 73.8% were female (*n* = 516). Regarding education, 28.18% (*n* = 197) completed primary/middle school, 16.01% (*n* = 114) completed high school, and 55.51% held a bachelor’s or master’s degree. The majority of the sample was working (67.00%, *n* = 468), 12.73% were students (*n* = 89), 11.87% were retired (*n* = 83), 5.72% (*n* = 40) were homemakers, and 22.58% (*n* = 18) were unemployed. Among the entire sample, 32.9% (*n* = 230) endorsed the full criteria of AjD according to ICD-11.

#### Groups comparison

Groups did not differ in age and gender. In both the Swiss (73.8%) and the UK 64.1%) samples, there were more women than men (*p* = 0.212). Age also did not differ significantly between groups (*p* = 0.583). In both groups, the proportion of retired and unemployed participants was similar.

#### Measurements

The ADNM-8 [12] assesses the preoccupation and failure to adapt similarly to the ICD-11. Participants first rate a list of stressors, indicating which stressors they experienced during the previous 2 years. Then, they rate the presence of AjD symptoms during the last 2 weeks. Four items refer to preoccupation with the stressor(s) and four items assess failure to adapt symptoms (see Table 2). Each item is scored on a 4-point Likert-type scale (1 = never, 2 = rarely, 3 = sometimes, 4 = often). The total score of the ADNM-8 is the sum of responses to all items, and higher scores are indicative of greater severity of AjD. The internal reliabilities (Cronbach’s alphas) of the ADNM-8 were satisfactory for the UK (0.812) and Swiss (0.850) samples for the total scores as well as for the preoccupation and the failure to adapt subscales in the UK (0.686, 0.780) and Switzerland (0.816, 0.711), respectively.

**Table 2.** ADNM items.

Preoccupation	Clinical mean (SD)	Nonclinical mean (SD)
Item 1: I have to think about the stressful situation repeatedly	3.51 (0.80)	3.31 (0.79)
Item 2: I have to think about the stressful situation a lot and this is a great burden to me	3.23 (0.98)	2.46 (0.92)
Item 4: I constantly get memories of the stressful situation and cannot do anything to stop	3.28 (0.96)	1.78 (0.93)
Item 5: My thoughts often revolve around anything related to the stressful situation	3.32 (1.38)	2.14 (0.89)
Failure to adapt		
Item 3: Since the stressful situation, I find it difficult to concentrate on certain things	3.40 (0.85)	1.79 (0.91)
Item 6: Since the stressful situation, I do not like going to work or carrying out necessary tasks in everyday life	2.95 (1.09)	1.85 (0.97)
Item 7: Since the stressful situation, I can no longer sleep properly	3.36 (0.97)	1.58 (0.87)
Item 8: Overall, the stressful situation affected me strongly in my personal relationships, my leisure activities, or in other important areas of life	3.44 (0.86)	2.78 (1.05)

#### Statistical analysis

We analyzed the symptoms network of ICD-11 AjD using the ADNM-8 in a clinical dataset from the UK, as compared to the network structure of ADNM-8 in a nonclinical sample from Switzerland.

#### Regularized partial correlation networks across the three samples

More information regarding network estimation and stability and accuracy of both edges and the centrality index techniques can be found in the data analysis section of the Supplementary Materials.

#### Network estimation and visualization

We estimated partial pairwise correlations parameters between all nodes, through a Gaussian graphical model. The methodology is described in detail in the data analysis section of the Supplementary Materials. We used the graphical least absolute shrinkage and selection operator (Graphical Lasso; implemented in qgraph), which visualizes sparse networks using part correlations and considered the ordinal scale of the questionnaire.

#### Network inference

The centrality index node strength and the predictability of each node. Strength refers to the sum of all edges connected to a specific node [22]. Strength provides information on the connectedness of each node within the symptom network and it is considered a relative metric.

#### Network stability

We examined the stability of the individually estimated networks, including estimating 95% confidence intervals around the edge weights and estimating a correlation-stability coefficient for strength centrality. More information regarding the network analysis techniques can be found in the data analysis section of the Supplementary Materials and in a tutorial [23].

#### Network comparisons

To compare differences between networks, we estimated network differences between each pair of networks using the *NetworkComparisonTest* (NCT) package in R [24]. More information regarding the network comparisons techniques can be found in the data analysis section of the Supplementary Materials.

## Results

### Regularized partial correlation networks across the two samples

#### Network estimation

**Figure 1.**
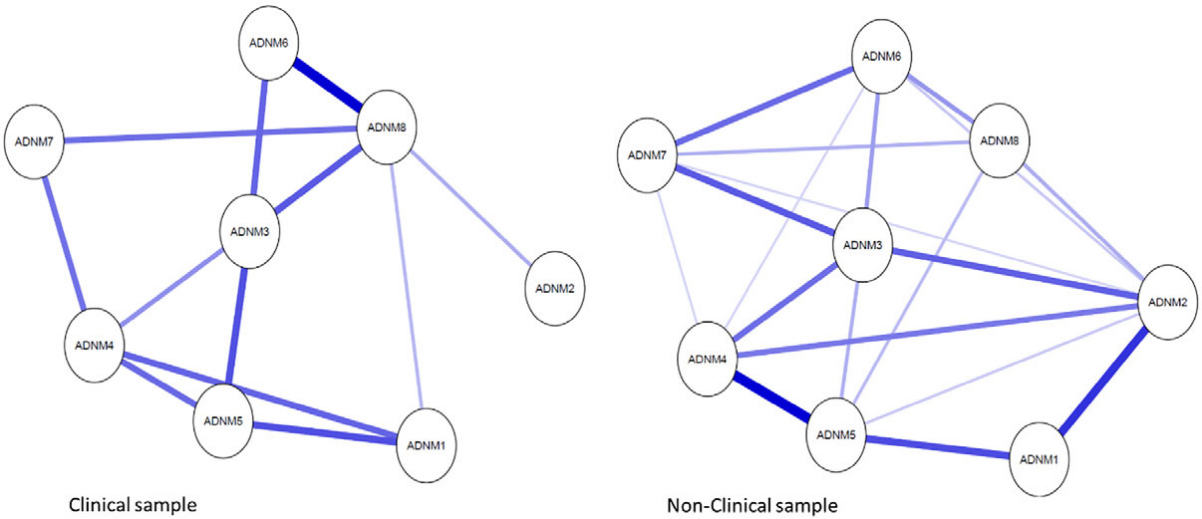
Networks of ADNM-8 adjustment disorder symptoms in clinical versus nonclinical datasets using average spring layout. Nodes represent ADNM-8 items, and edges regularized partial correlations with LASSO penalty. Distances among nodes and thickness of edges relate to the size of their partial correlations. Blue edges indicate positive relations and red edges indicate negative relationships. ADNM 1, repeated thoughts; ADNM 2, sense of burden; ADNM 3, difficulties concentrating; ADNM 4, constant memories; ADNM 5, thoughts revolve; ADNM 6, work/tasks difficulties; ADNM 7, sleeping problems; ADNM 8, functional impairment. The full items can be found in Table 1.

To enhance visual comparability of edges, we estimated the average layout of the two networks and presented all networks using this layout (Figure 1). In the clinical sample network, 12 of 28 possible edges (42.9%) while 19 of 28 possible edges (67.9%) in the Swiss nonclinical network, were nonzero. This designates that the symptoms had extensive connections with each other in both samples. The visual inspection of the networks exhibited many inconsistent edges across the samples.

#### The ADNM-8 symptoms network in the UK clinical sample

In the clinical network, the most robust connection was found between the “difficulties doing work/tasks” (item 6) and the impairment in functioning item (item 8), both represent the failure to adapt factor. Next in the hierarchy of edges strength is the association between the “repeated thoughts” (item 1) and 5 (“thoughts often revolve”) both belong to the preoccupation factor. Then, strong associations were found between the preoccupation items 4 (“constant memories”) and 5 (“thoughts often revolve”), as well as between item 4 and item 1 (“repeated thoughts”). Equal strength of association was found between item 3 (“difficulties concentrating”) which is a part of the failure to adapt factor and 5 (“thoughts often revolve”) which is a part of the preoccupation factor. Weaker but yet significant associations were found between item 3 (“difficulties concentrating”), on the one hand, and the “difficulties doing work/tasks” (item 6) and the impairment in functioning item (item 8) of the failure to adapt factor, on the other hand. The “sense of burden” (item 2) was distant from all other symptoms and weakly connected to the network.

#### The ADNM-8 symptoms network in the Swiss nonclinical sample

The strongest association was found between the preoccupation items 4 (“constant memories”) and 5 (“thoughts often revolve”), followed by a robust connection between the preoccupation items: “repeated thoughts” (item 1) and “sense of burden” (item 2). Next in the hierarchy of edges strength was the association between the preoccupation items 1 (“repeated thoughts”) and 5 (“thoughts often revolve”). Then, there were strong connections between the failure to adapt item 7 (“sleep difficulties”) on the one hand and items 3 (“difficulties concentrating”) and 6 (“difficulties going to work/doing daily tasks”) on the other hand. Equally strong was the association between items 2 (“sense of burden”) and 3 (“difficulties concentrating”). Finally, there were strong associations, though less substantial between the preoccupation “sense of burden” (item 2) and item 4 (“constant memories”). Item 4 was equally associated with item 3 (“difficulties concentrating”) of the failure to adapt factor. Item 8 was less connected to other symptoms in the network.

#### Network stability

To confirm the visual similarity of networks, we used Spearman correlations of edge weights for all combinations of networks, which are presented in the data analysis section of the Supplementary Materials. Analysis shows that the accuracy of the edges was satisfactory. The results of the confidence interval showed that edge weights were moderately large. In addition, the results showed moderate accuracy of the centrality strength index (see Supplementary Material text, results: Network accuracy and stability and Supplementary Figures S2–S3).

#### Network inference

**Figure 2.**
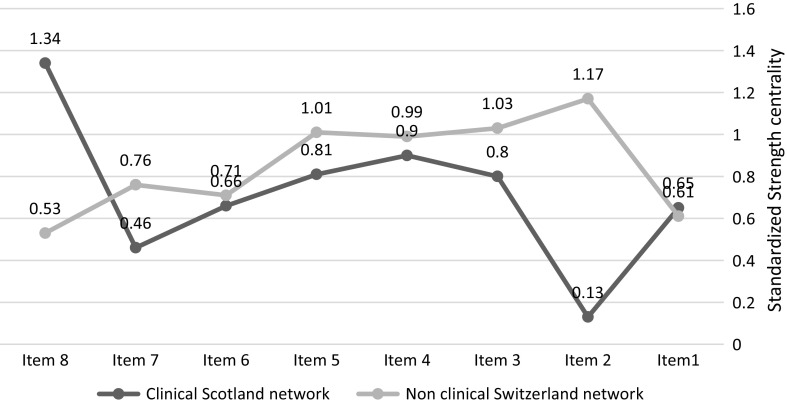
Standardized node strength centrality for the networks. ADNM 1, repeated thoughts; ADNM 2, sense of burden; ADNM 3, difficulties concentrating; ADNM 4, constant memories; ADNM 5, thoughts revolve; ADNM 6, work/tasks difficulties; ADNM 7, sleeping problems; ADNM 8, functional impairment. The full items can be found in Table 1.

The standardized strength centrality estimates are presented in Figure 2. Item 2 (“sense of burden”) was the node with the highest strength centrality in the nonclinical sample’s network. The node with the smallest centrality was the impairment in functioning (item 8). In the clinical sample’s network, the node with the highest centrality was the impairment in functioning item 8 and the least central item was item 2 (“sense of burden”).

#### Network comparisons

Results from the network comparison test showed that global strength values per group were 3.41 and 2.87 for Scotland (clinical data) and Switzerland (nonclinical data), respectively (S statistics was 0.53 and *p* value was 0.14). The network structure differed between the two samples (*M* = 0.33, *p* = 0.03).

## Discussion

This was the first study to compare the symptom network structure of the ICD-11 AjD in a clinical sample compared to a nonclinical sample. In both samples, extensive connections were found between the symptoms with particularly strong associations within each core symptom cluster (i.e., preoccupation with the stressor and failure to adapt symptoms). While global strength was similar between networks, the networks’ structures differed. In the clinical network, the most robust connections were found between items representing failure to adapt, including the “impairment in functioning” item. Conversely, in the nonclinical sample, the strongest associations were found between preoccupation items. Regarding centrality of symptoms, in the clinical sample’s network, the node with the highest centrality was “impairment in functioning” and the least central item was the preoccupation item “sense of burden.” Interestingly, the nonclinical sample showed the opposite trend, with “sense of burden” being the most central item while the “impairment in functioning” being the least central item.

### Conceptual validity and central symptoms

This study aimed to assess the conceptual validity by exploring which of the symptoms strongly associate with one another and are located adjacently and to identify and compare the most central symptoms in the networks. Our findings indicate that the global strength of the clinical and nonclinical networks was similar. This finding lends support to the overall intensity of connections (weighted absolute sum of all edges) between symptoms in both networks of ICD-11 AjD, which indicates that the overall strength of associations between symptoms in the two networks was similar. Specifically, in both samples, extensive connections were found among the symptoms within each core symptom cluster (i.e., preoccupation with the stressor and failure to adapt). This provides further evidence for the conceptual validity of this newly defined condition.

However, the structure of the networks of the clinical and nonclinical samples and the most central symptoms differed significantly. In the nonclinical sample, it was found that preoccupation symptoms were the most connected nodes in the AjD network and that the preoccupation item “sense of burden” was the node with the highest strength centrality. The node with the lowest centrality was “impairment in functioning.” This is in line with a recent study that conducted symptoms network analysis among three population-based African samples and found similarly strong associations between preoccupation symptoms [18], supporting the notion that preoccupation is the most prominent indicator of AjD in nonclinical samples. Moreover, in the population-based African samples, the “sense of burden” item also had the highest strength centrality in all three nonclinical networks [18]. This could be explained by the fact that failure to adapt symptoms is more heterogeneous than preoccupation symptoms, describing symptoms such as sleep disturbances, concentration difficulties, loss of interest in positive activities, and reduced self-confidence [25].

Interestingly, among the clinical cases, for whom the adjustment difficulties are more substantial, failure to adapt items was more intensively connected and “impairment in functioning” played a distinctive role. The least central item was the preoccupation item “sense of burden,” which was the most central item in the nonclinical sample. Results indicate that in the clinical sample impairment in functioning was a core symptom of the disorder. While functional impairment represents global malfunctioning in broader domains of life, the specific failure to adapt symptoms can be understood as subjective difficulties with work/tasks and other related psychopathological variables such as sleeping problems and concentration difficulties. The high centrality of failure to adapt in respect to functioning is also consistent with other studies conducted with clinical samples. For example, the symptoms network research conducted among clinical samples with schizophrenia showed that functioning and difficulties with tasks of daily life were most central and highly interconnected nodes in networks of schizophrenia [26].

The pattern of the current results raises the tentative assumption that there may be temporal development according to which AjD is first characterized by emerging preoccupation symptoms and less substantial failure to adapt symptoms (i.e., the pattern prevalent in nonclinical populations). If the preoccupation symptoms persist, it could be assumed that they result in failure to adapt and functional impairment, which represents the clinical manifestation of AjD (i.e., the pattern prevalent in clinical populations). This assumption is in line with the theoretical stress response model of Horowitz [27], which proposes that AjD can be located on a stress response continuum, along with other stress response syndromes such as Post traumatic stress disorder (PTSD) and prolonged grief. The model proposes four consecutive phases of stress response, starting with a first phase of realization that is accompanied by negative emotions such as fear, sadness, or rage. The second phase is characterized by denial and refusal to face the implications of the event, which in the third phase results in alternating intrusions (i.e., preoccupations) and suppression of these unbidden thoughts and memories. In its final fourth phase, the stress response process results either in adapting to the stressor and its consequences or in problems to adapt, the latter of which represent a mental disorder such as AjD.

In line with this model, it was recently argued that preoccupation may represent a generic risk factor for the development of psychopathology [28]. The current study lends further support for the transdiagnostic potential of preoccupation. Preoccupation may provide the grounds for the development of AjD which gradually enables the development of failure to adapt and functioning difficulties as observed in clinical cases. This hypothesis, however, requires further investigation in longitudinal studies with multiple assessments.

The study has several limitations. First, even though both samples are Western-European, we cannot negate the alternative explanation that cultural differences are associated with differences between the samples. Second, the data collected relied on a self-report measure rather than clinician-administered interviews, which may have biased the reports. Third, the cross-sectional nature of the data does not allow for any inferences on causality. Moreover, the centrality measures may be high because the nodes strongly influence the rest of the system but also because they are influenced by other nodes. Fourth, the stressor referred to when administering the ADNM was different in the two samples (the COVID-19 pandemic in Switzerland; general stressors in the UK). This discrepancy renders the samples not directly comparable. Fifth, the Swiss sample is a convenience sample and, therefore, is not representative of the Swiss population. Despite these limitations, the current findings provide an initial estimation of the network structure of AjD in clinical and nonclinical samples with important insights that can guide future research and practice.

### Clinical implications

Comparing the symptom networks of a clinical and a nonclinical sample provided some initial thoughts regarding the temporal development of the disorder which also have implications for psychosocial interventions. This is particularly important considering that relatively little is known about the treatment of AjD. It has been suggested that adjustment difficulties after stressful life events should be addressed with a stepped care approach [29], whereby low-intensity interventions such as bibliotherapy, behavioral activation, and e-mental-health interventions are suitable in the early stress response stage [25]. The results of the current study suggest that exercises focused on handling preoccupation could be useful as preventive measures and potentially inhibit later failure to adapt symptoms as well as the full clinical picture of AjD. Specifically, psychoeducation in a preventive setting should portray preoccupation as a natural process that supports the integration of the life event into one’s biography. Supportive measures should guide clients to reprocess and understand their experiences in a meaning-making process. Clients must not develop avoidance strategies because attempts to suppress distressing thoughts about the event are often doomed to failure and can perpetuate the disorder in the long term [28, 30].

The prominence of functional impairment in the clinical sample’s network suggests prioritizing failure to adapt and functional capacity as treatment targets for AjD in clinical settings. The high centrality of functional impairment in the network supports approaches to AjD that consider the ability to perform daily tasks in everyday life as a primary target of recovery programs [29]. A failed adjustment to the stressor is often accompanied by feelings of personal incompetence and thus a resource-strengthening approach is indicated. It can be useful to guide patients to recall past crises and to identify personal qualities and strengths that helped them deal with difficult situations in the past. Subsequently, it should be elaborated on how these resources can be used purposefully in the current life situation [30, 31]. Furthermore, if patients report sleep and concentration issues and a limited ability to recover, sleep, hygiene, as well as a balance between activity and relaxation, can counteract these problems [31].

## Data Availability

The data that support the findings will be available upon request from the corresponding author.

## Supplementary material

For supplementary material accompanying this paper visit https://doi.org/10.1192/j.eurpsy.2022.2303. click here to view supplementary material
