# Supplementary material for: The network structure of ICD-11 adjustment disorder: A comparison of clinical and nonclinical samples
Source: Eur Psychiatry. 2022 Jul 29;65(1):e43. doi: 10.1192/j.eurpsy.2022.2303 (PMC9393912; doi:10.1192/j.eurpsy.2022.2303)
Supplement: Supplementary file 1 [file S0924933822023033sup001.docx]

**Data analysis**

This section provides am more detailed information about the statistical procedure and the used software. Network stability and accuracy was measured by methods previously described in details (1). We used R-package *bootnet* to establish robustness (2).

**Network estimation**

We estimated Gaussian Graphical Models (GGM) for pairwise association parameters between all nodes. In the GGM, edges can be understood as conditional dependence relations among symptoms: If two symptoms are connected in the resulting graph, they are dependent after controlling for all other symptoms. Symptoms that are not connected are conditionally independent. With 8 symptom nodes, 28 pairwise association parameters are estimated. The estimation of so many parameters may lead to a number of spurious connections; we thus controlled for these false positives by using the least absolute shrinkage and selection operator (LASSO; 1) which sets very small edges to zero. This procedure employs a regularization technique that conservatively identifies only the relevant edges, and accurately discovers the underlying network structure (4). More details on these estimation techniques, including a tutorial, is available elsewhere (5).

The symptom network was estimated for all symptoms of the ADNM-8 using the R-package qgraph (6). Networks were estimated using regularized partial correlation models in the R-package qgraph that present the unique, independent relationships between symptoms (7). The network is weighted and undirected due to the cross-sectional nature of the study. Questionnaire data of Adjustment disorder symptoms (8-item ADNM version) were answered at an ordinal scale; thus, we estimated a matrix of polychoric correlations.

**Visualization with the Graphical Lasso** This method directly estimates the inverse of the covariance matrix (8). It shrinks small edges and many parameters to zero by estimating a penalized [maximum likelihood](https://www-sciencedirect-com.ezprimo1.idc.ac.il/topics/medicine-and-dentistry/maximum-likelihood-method) solution based on the Extended [Bayesian Information Criterion](https://www-sciencedirect-com.ezprimo1.idc.ac.il/topics/medicine-and-dentistry/bayesian-information-criterion) (EBIC) (9). For ease of visual comparison, the networks were restricted to a consistent “average layout,” presented for the two samples. The networks are presented in figure SM1 without "average layout" while figure 1 in the manuscript were restricted to a consistent “average layout.

**Network stability**

We performed two procedures to check the accuracy and stability of the results: (A) the accuracy of estimated edge-weights (figure SM2), (B) the stability of centrality indices (figure SM3) after subsetting the data (10). The CS coefficient is defined as the maximum proportion of cases that can be dropped, such that with 95% probability the correlation between the original centrality metric and the centrality of networks based on bootstrapped subsets is 0.7 or higher (10). These authors also suggest interpreting CS coefficients of 0.5 or larger as preferable, and CS coefficients of 0.25 or larger as the minimum requirement.

There are no clear boundaries to interpret the results of the stability analyses. The confidence intervals around the edge weights were moderately large, indicating a moderate accuracy of the network estimation (Figure SM2). Most edge weights overlapped; however, the largest edges did not include zero and less overlapped with most other edges in the network (see Figure SM2). Figure SM3 displays the edges that significantly differ from each other. The correlation of the original strength centrality order with the order of the strength centrality in subsets is moderate, even after dropping a substantial number of participants (Figure SM3), which means that the strength estimate can be considered moderately stable, in particular in the Swiss sample, though in both samples the stability was satisfactory.

To test the stability of the metrics, we used the CS coefficient (10) and interpreted values below 0.25 as unacceptable. The samples had sufficient stability of the edges across imputations, for the clinical sample from Scotland (0.361 CI 95% 0.285, 0.439) and closer to even strong stability for the non-clinical sample from Switzerland (0.594 CI 95% 0.517, 0.672). The correlation stability coefficient for the strength centrality metric above the suggested threshold of 0.25 for moderate stability (10) for both the samples for Switzerland (.361 CI 95% 0.283, 0.439) and Scotland (0.439 CI 95% .361, .518). This indicates that the centrality strength indexes are moderately stable under subsetting cases in samples. Yet, the centrality index should be referred cautiously.

Figure SM4 displays the centrality estimates of all eight items that significantly differ from each other. and Figure SM5 displays the centrality estimates of all eight items that significantly differ from each other.

**Network Comparisons**

Overall connectivity can be summarized by global strength and is defined as the weighted absolute sum of all edges in the network. The distance S, based on global strength, between two networks is presented. The Invariant Network Structure concerns the structure of the network as a whole. The test of network structure invariance evaluates the observed value of M in the data against the reference distribution of M that arises from random permutation of group membership across cases (11).

**Table S1.**

*ADNM items*

| ADNM items |
| --- |
| Preoccupation |
| Item 1: I have to think about the stressful situation repeatedly |
| Item 2: I have to think about the stressful situation a lot and this is a great burden to me |
| Item 4: I constantly get memories of the stressful situation and can’t do anything to stop |
| Item 5: My thoughts often revolve around anything related to the stressful situation |
| Failure to adapt |
| Item 3: Since the stressful situation, I find it difficult to concentrate on certain things |
| Item 6: Since the stressful situation, I don’t like going to work or carrying out necessary tasks in everyday life |
| Item 7: Since the stressful situation, I can no longer sleep properly |
| Item 8: Overall, the stressful situation affected me strongly in my personal relationships, my leisure activities, or in other important areas of life |


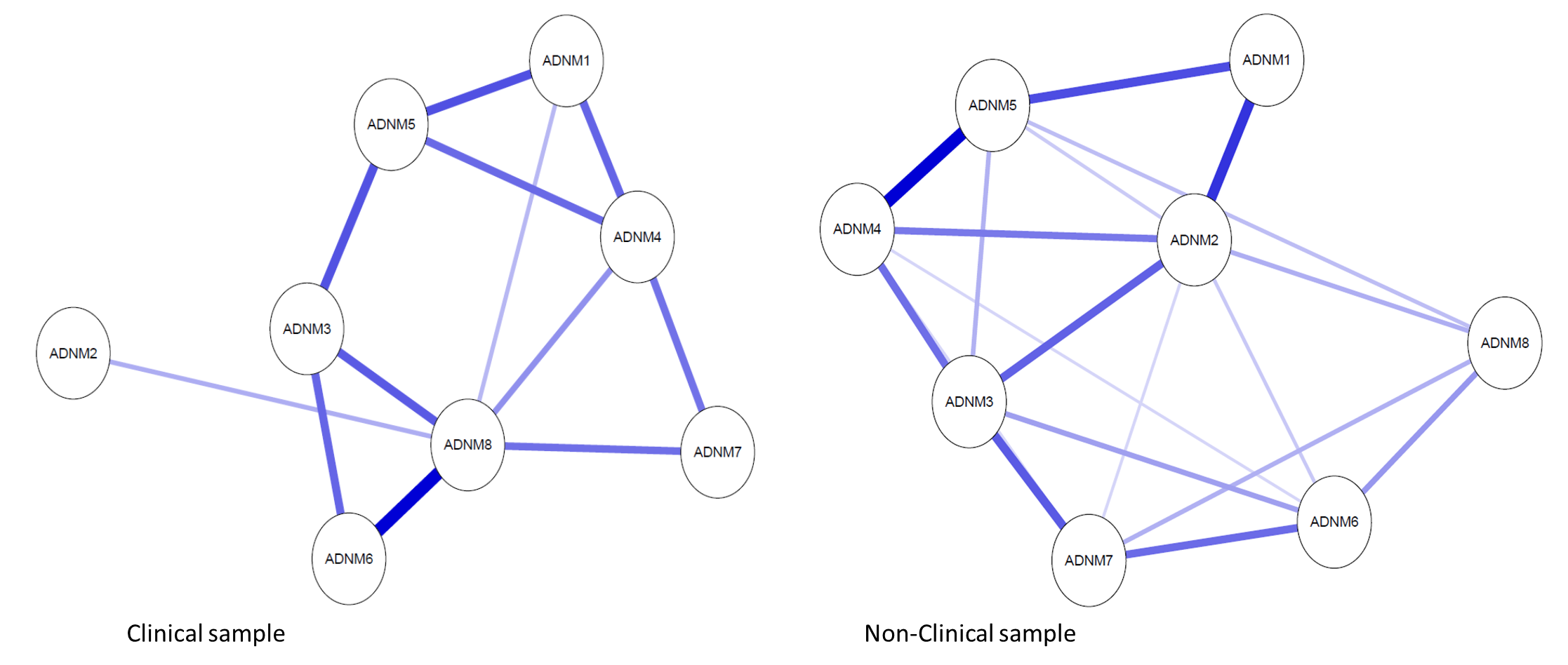


**Fig SM1.** Networks of ADNM-8 adjustment disorder symptoms in clinical vs. non-clinical datasets. Nodes represent ADNM-8 items, and edges Regularized partial correlations with LASSO penalty. Distances among nodes and thickness of edges relate to the size of their partial correlations. Blue edges indicate positive relations and red edges indicate negative relationships. ADNM 1: Repeated thoughts, ADNM 2: Sense of burden; ADNM 3: Difficulties concentrating; ADNM 4: Constant memories; ADNM 5: Thoughts revolve; ADNM 6: Work/tasks difficulties; ADNM 7: Sleeping problems ADNM 8: Functional Impairment. The full items can be found in Table S1.

**
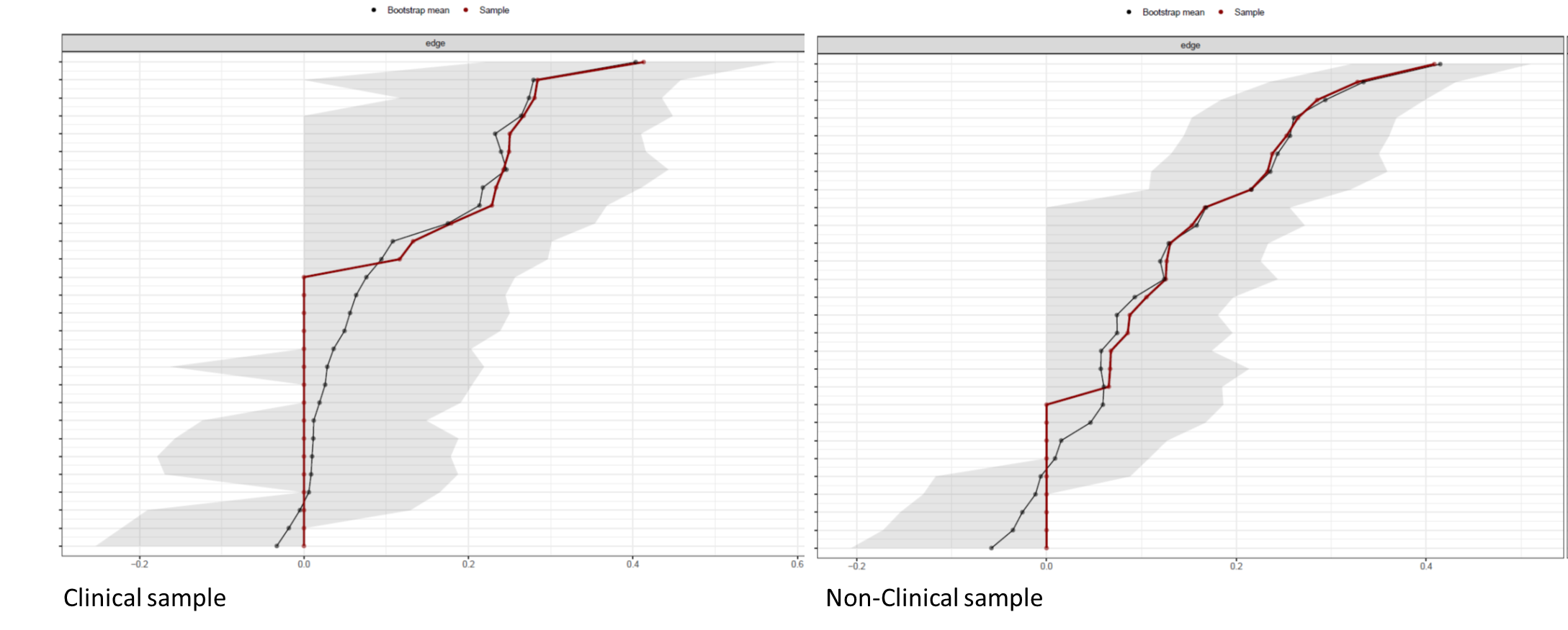
**

**Fig SM2.** Stability analysis – ADNM-8 - accuracy of edge weights. Red lines are Point estimates and grey are the 95% bootstrap confidence intervals of network edges (based on partial correlations between items) for the estimated networks.

**
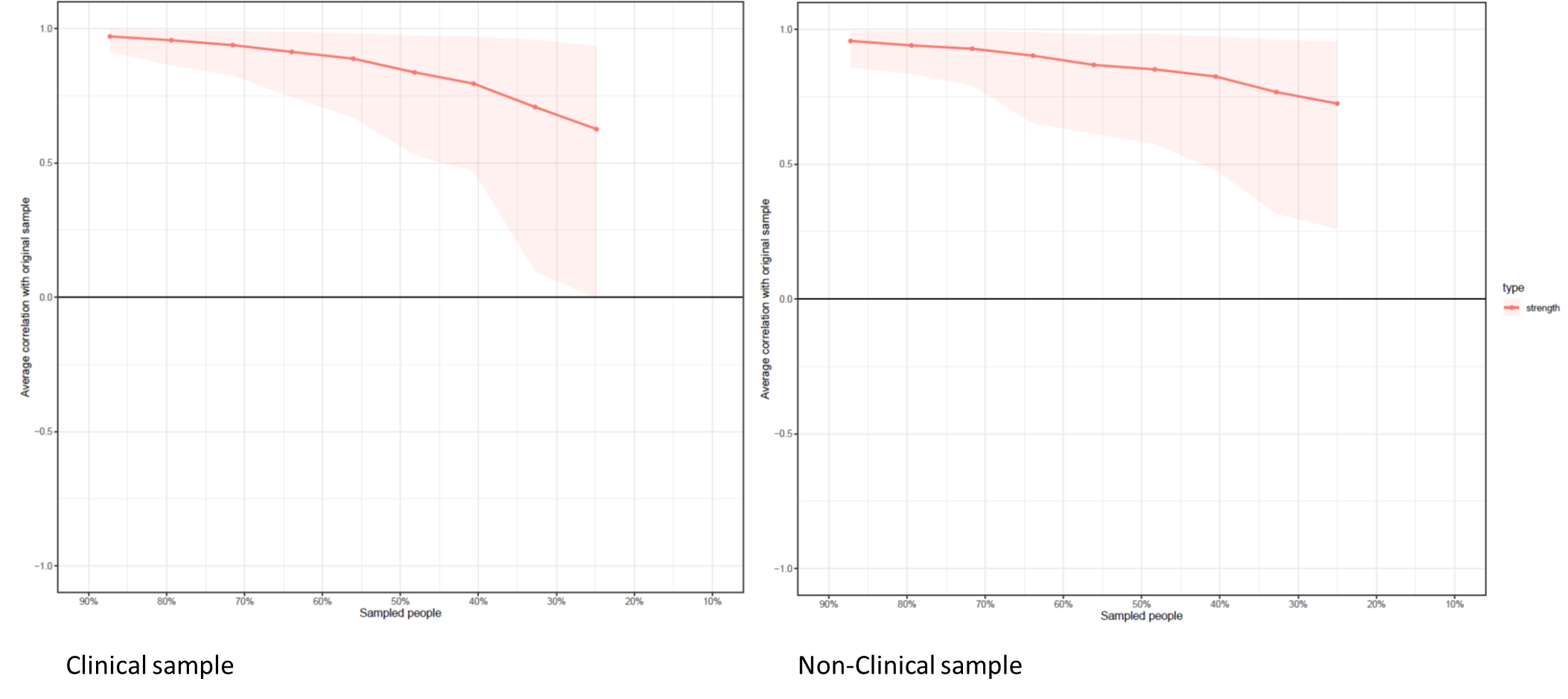
**

**Figure SM3. Stability Analysis**. **ADNM-8 -** Centrality bootstrap. Correlation of the original strength centrality order with the order of strength centrality in subsets of the data. The correlation after dropping a substantial number of participants is high for the centrality metric strength, which means that this centrality estimate can be considered stable in all three samples.

**
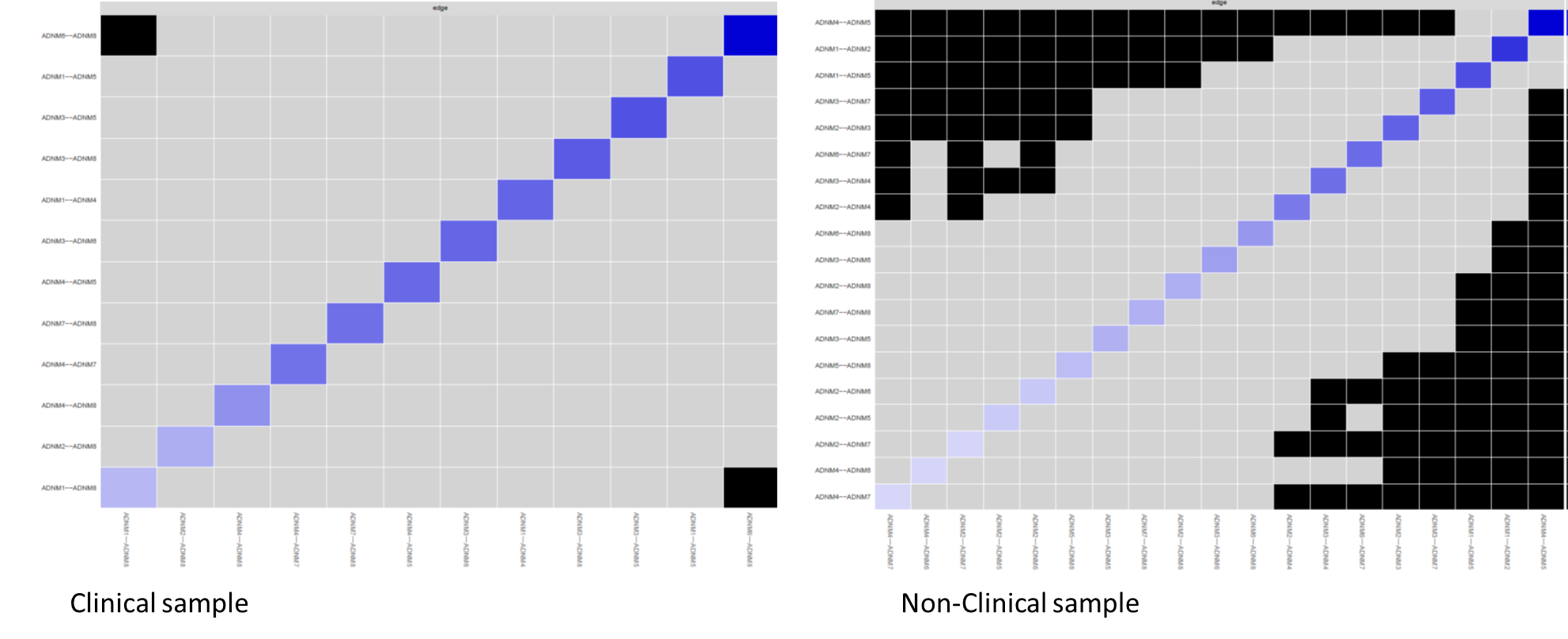
**

**Figure SM4. Edge weights difference test.** **ADNM-8 -** Black boxes represent significant differences between edge weights. The test does presently not correct for multiple testing.

| 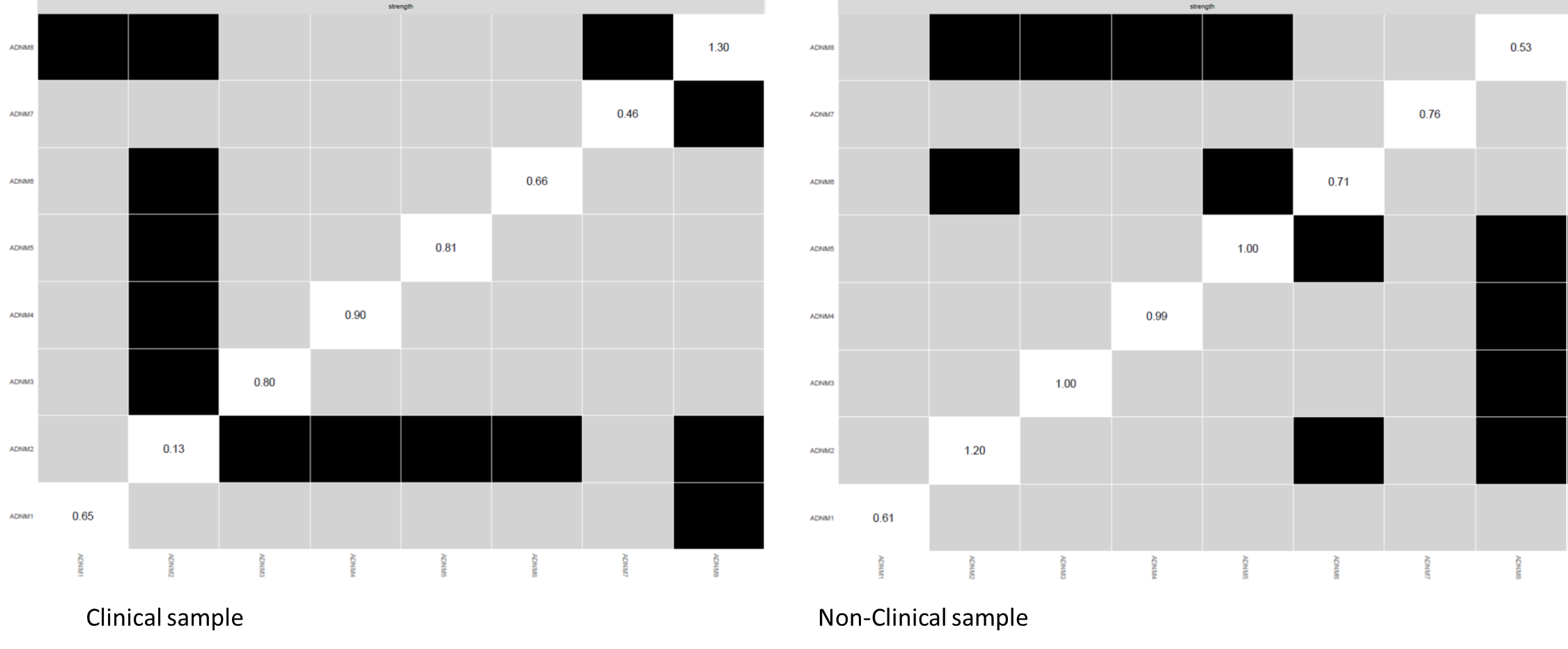  **Figure SM5. Centrality difference test.** Standardized strength centrality values are shown in the diagonal, black boxes represent significant differences in centrality estimates. The test does presently not correct for multiple testing.  References  1. Knefel M, Karatzias T, Ben-Ezra M, Cloitre M, Lueger-Schuster B, Maercker A. The replicability of ICD-11 complex post-traumatic stress disorder symptom networks in adults. Br J Psychiatry 2019; **214**(6): 361-368. Available from: <https://doi.org/10.1192/bjp.2018.286> 2. Epskamp S. Package ‘bootnet’: Bootstrap Methods for Various Network Estimation Routines; 2015. Available from: URL: <https://cran.r-project.org/web/packages/bootnet/index.html>.  3. Friedman J, Hastie T, Tibshirani R. Sparse inverse covariance estimation with the graphical lasso. Biostatistics 2008; 9(3):432–41.  4. van Borkulo CD, Borsboom D, Epskamp S, Blanken TF, Boschloo L, Schoevers RA et al. A new method for constructing networks from binary data. Sci Rep 2014; 4:5918.  5. Epskamp S, Fried EI. A tutorial on regularized partial correlation networks. Psychol Methods 2017. Available from: URL: <https://arxiv.org/abs/1604.08462>.  6. Epskamp S, Cramer AOJ, Waldorp LJ, Schmittmann VD, Borsboom D. Qgraph: Network visualizations of relationships in psychometric data. *J Stat Softw* 2012; **48**: 1–8.  7. Knefel M, Karatzias T, Ben-Ezra M, Cloitre M, Lueger-Schuster B, Maercker A. The replicability of ICD-11 complex post-traumatic stress disorder symptom networks in adults. *Br J Psychiatry* 2019; **204**: 361–8.  8. Friedman J, Hastie T, Tibshirani R. Sparse inverse covariance estimation with the graphical lasso. *Biostatistics* 2008; **9**: 432–41.  9. Foygel R, Drton M. Extended Bayesian information criteria for Gaussian graphical models. In *Advances in Neural Information Processing Systems 23: 24th Annual Conference on Neural Information Processing Systems 2010, NIPS 2010*: 2020–8. , 2010.  10. Epskamp S, Borsboom D, Fried EI. Estimating psychological networks and their accuracy: A tutorial paper. Behav Res Methods 2017.  11. van Borkulo CD, Boschloo L, Kossakowski JJ, Tio P, Schoevers RA, Borsboom D, *et al.* Comparing network structures on three aspects: A permutation test. , 2017 (http://dx.doi.org/10.13140/RG.2.2.29455.38569). |
| --- |
